# Concurrent ruptured spontaneous heterotopic pregnancy and ruptured appendix with delayed presentation in the first trimester: a case report

**DOI:** 10.11604/pamj.2020.37.222.26182

**Published:** 2020-11-05

**Authors:** Grant Murewanhema, Simbarashe Madombi, Lynette Hlathswayo, Ndabaningi Simango

**Affiliations:** 1 Department of Obstetrics and Gynaecology, College of Health Sciences, University of Zimbabwe, Harare, Zimbabwe

**Keywords:** Case report, heterotopic pregnancy, acute appendicitis, ruptured appendix

## Abstract

Acute appendicitis is the commonest non-gynaecological surgical emergency in pregnancy. However, the concurrent occurrence of acute appendicitis with a heterotopic pregnancy is a rare event and presents diagnostic challenges to unsuspecting clinicians and sonographers. We present a case of a woman who had a heterotopic pregnancy and was noted to have a gangrenous appendicitis at laparotomy, illustrating how a diagnosis of acute appendicitis could easily be missed in pregnancy. We report the case of a 34-year-old woman in the first trimester of pregnancy who had a missed diagnosis of acute appendicitis after she had complained of vague abdominal symptoms for three weeks. She presented to a gynaecologist with vaginal bleeding for three days and was noted to have a heterotopic pregnancy on ultrasound scan. At laparotomy, she was noted to have a gangrenous appendicitis with pyoperitoneum concurrent with a ruptured left fimbrial ectopic pregnancy. Left salpingectomy and saline lavage were done and she had uneventful post-operative recovery. Unsuspecting clinicians, in patients without risk factors, can miss both heterotopic pregnancy and acute appendicitis. As assisted reproductive techniques become widespread, the possibility of heterotopic pregnancies must always be considered and any patient who presents with vague abdominal symptoms must be suspected to have the possibility of acute appendicitis. Because of the unreliability of laboratory investigations and clinical predictive scores in pregnancy, sonographers must be specifically asked to scan for heterotopic pregnancy and appendicitis in suspected cases.

## Introduction

This case has been reported according to the CARE guidelines [1]. Acute appendicitis is the commonest non-gynaecological surgical emergency in pregnancy, with an incidence of 1 in 800-1500 pregnancies [2-6]. About 30% of cases occur in the first trimester, 45% in the second trimester and the remaining 25% in the third trimester [2,7,8]. Lymphoid hyperplasia accounts for the majority of cases, whilst faecoliths and other causes are responsible for the rest [9]. Anatomically, as the pregnancy progresses, the position of the appendix is displaced upwards from the right iliac fossa (RIF) by the gravid uterus [2]. Some of the symptoms of early pregnancy including nausea, anorexia and abdominal discomfort and those of acute appendicitis mimic each other, leading to delays in diagnosis and intervention for acute appendicitis in pregnancy [8,10,11].

A heterotopic pregnancy (HP) signifies the simultaneous occurrence of an extra-uterine and an intrauterine pregnancy (IUP). The occurrence is very rare with spontaneous pregnancies, estimated to be about 1:30,000 pregnancies [12,13]. Heterotopic pregnancies have a higher incidence of about 1:3900 pregnancies with assisted reproduction technologies (ART) such as in-vitro fertilization (IVF) and gamete intra-fallopian transfer (GIFT) [13,14]. The use of clomiphene citrate for ovulation induction could also increase the incidence [12]. Other risk factors include tubal damage from previous pelvic inflammatory disease (PID), history of ectopic pregnancy, miscarriage and ovarian hyper-stimulation syndrome [14,15].

The concurrent occurrence of an ectopic pregnancy with acute appendicitis is a rare event, with no sufficient data to estimate the incidence [16]. Rarer is the concurrent occurrence of an HP with acute appendicitis [17]. Such cases present diagnostic and therapeutic challenges for clinicians and are associated with increased morbidity and mortality [16,17]. We present a rare case of a ruptured appendix with delayed presentation which was noted at laparotomy, in a patient with a heterotopic pregnancy.

## Patient and observation

**History:** a 34-year-old woman in her third pregnancy at an estimated gestational age of 11 weeks, with both her children alive, presented to our hospital. She had started experiencing mild, poorly localised abdominal pain about three weeks prior to presentation and the day before presentation she started bleeding vaginally, with associated mild backache in addition to the pre-existing abdominal pain. The bleeding was light and she did not develop any symptoms for significant volume loss. She presented to her gynaecologist, who ordered a pelvic ultrasound scan (USS) as part of the evaluation. On USS, an HP was diagnosed, with a single viable intrauterine foetus and a demised left adnexal foetus and she was referred to our hospital for further management. A systemic enquiry was done which was unremarkable with no constitutional symptoms. She did not have any significant gastro-intestinal and genitourinary symptoms. She had delivered vaginally in her two previous pregnancies and had not experienced any complications. She was HIV negative, had no previous history of treatment for sexually transmitted infections and had not been screened for cervical cancer before. She did not have any significant medical and surgical history. She was of sober habits, unemployed and stayed with her partner in rented accommodation in a high-density suburb and was not on medical insurance.

**Figure 1:**
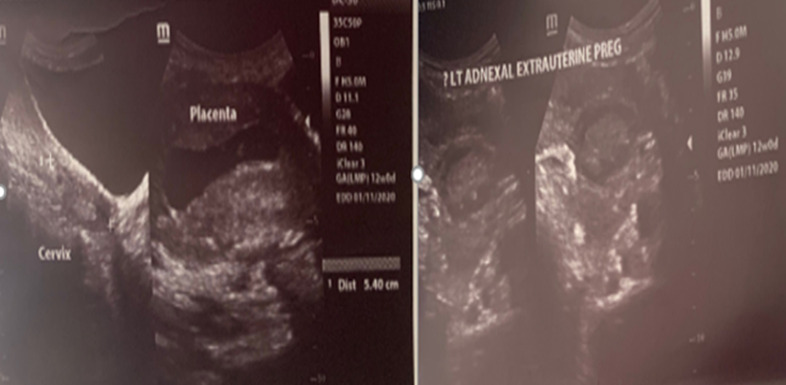
ultrasound scan images showing an intrauterine pregnancy and a left-sided ectopic pregnancy

**Physical examination:** she was fully conscious and hemodynamically stable, with a blood pressure of 123/87 mmHg and a pulse rate of 90 beats per minute. Her temperature was 36.7 degrees Celsius, however, she felt hot to touch. She had mild central pallor. The abdominal examination was unremarkable. The uterus was not palpable above the symphysis pubis. She had bilateral adnexal fullness, no cervical motion tenderness and had no active vaginal bleeding or discharge. There were no significant findings in the respiratory, cardiovascular and central nervous systems. Her hemogram revealed a neutrophilic leucocytosis of 18.3 with a normocytic anaemia (Hb 9.0, MCV 83.0) (Figure 1).

**Figure 2:**
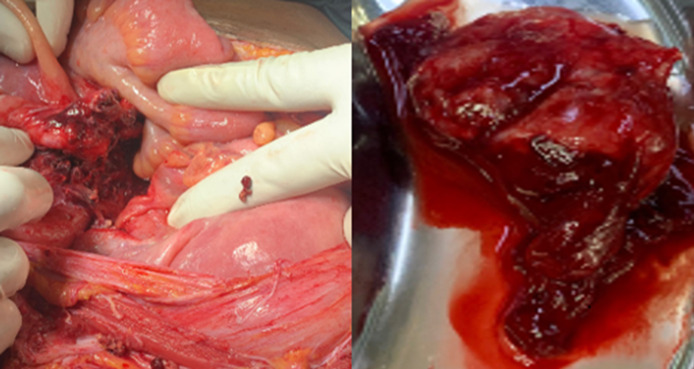
laparotomy findings; base of appendix showing total rupture after abdominal washout and left tubal ectopic pregnancy after salpingectomy

**Management:** we intended to take the patient for laparoscopy. However, due to theatre constraints we did a laparotomy. A Pfannestiel incision was fashioned and a pyoperitoneum of about 100 millilitres was encountered on entering the abdomen. We then did a midline incision to visualise all quadrants of the abdomen. A left-sided ruptured fimbrial ectopic pregnancy was noted and a left salpingectomy was done. An identifiable foetus was noted and not sent for histology. A ruptured appendix with a well-organised appendiceal abscess was also noted. The remnants of the appendix were minimal and all necrotic debris was removed. The rest of the abdomen was normal, with scanty fibrinous adhesions. A saline lavage was done prior to closure of the abdomen. Due to overwhelming necrosis, there was no appendix specimen to send for histology (Figure 2).

**Outcome:** post-operatively, the patient was managed on intravenous fluids, ceftriaxone, metronidazole and paracetamol and she recovered uneventfully. She was discharged on the fifth day post laparotomy. On follow-up review at two weeks patient was well and her scar was healing well. The pregnancy continued and was viable on USS at review, but the final outcome is still to be determined.

## Discussion

We have presented a rare and unusual case depicting the concurrent occurrence of a spontaneous heterotopic pregnancy and appendicitis. There is scarcity of literature describing such cases, more so in a patient without any identifiable risk factors, with no history of IVF or PID. Barnett *et al*. described a similar case of a ruptured HP and acute appendicitis in a patient who had undergone IVF [17]. Post-laparotomy the pregnancy progressed and patient went on to successfully deliver twins by caesarean section at term. Acute appendicitis presents diagnostic challenges in pregnancy, which may lead to delays in intervention, resulting in appendicular rupture, abscess formation, peritonitis, septicaemia and pregnancy wastage. Appendicitis is diagnosed clinically, aided by parameters of the Alvarado score and USS. The predictive Alvarado score may be unreliable in pregnancy owing to physiological changes in leucocytes and clinical parameters [18-20]. USS is an important imaging modality; however, in pregnancy appendicitis may be missed due to shift in position [2,6,20]. In a retrospective case analysis of 31 patients who underwent appendicectomy by Lin *et al*. 25 had pathologically confirmed appendicitis [11]. Abdominal ultrasonography had high sensitivity and specificity of 80% and 75% respectively, with an average diagnostic accuracy of 80.6% across the three trimesters. However, the study was limited by small sample size. Higher imaging modalities such as computed tomography and magnetic resonance imaging are generally not used due to cost limitations.

Appendicular rupture is more frequent in pregnant women due to diagnostic delays as in the case we presented and a general reluctance to operate on pregnant women [2]. Some obstetric and non-obstetric conditions, listed in Table 1, mimic acute appendicitis in pregnancy [2,8,19]. These conditions must be considered as possible differential diagnoses. In a literature review by Neto *et al*. it was noted that pregnant women were less likely to have a classical presentation of acute appendicitis [2]. The presented woman reported vague, poorly-localised abdominal pain. Pain around McBurney´s point is likely to occur in most pregnant women, regardless of the stage of pregnancy; however, in the third trimester, the pain may be located in the flank or right upper quadrant [2]. They recommended USS as an imaging modality of first choice, to avoid delays in diagnosis. A maximum diameter of greater than 6 mm of a tubular structure visualised in the RIF is diagnostic. A positive diagnosis on scan requires surgery. A sonographer must be specifically asked to examine the appendix; otherwise, the diagnosis can be missed.

**Table 1:** obstetric and non-obstetric conditions that can mimic acute appendicitis in pregnancy

Obstetric conditions	Non-obstetric conditions
Ectopic pregnancy	Acute pyelonephritis
Threatened miscarriage	Cholecystitis
Placental abruption	Acute pancreatitis
Preterm labour	Gastroenteritis
Round ligament pain	Renal calculus
Red degeneration of leiomyoma	Salpingitis
Chorioamnionitis	Mesenteric adenitis
Adnexal torsion	

In a hospital-based study on the management and outcomes of acute appendicitis in pregnancy, out of 56 pregnant patients admitted with suspected appendicitis, 51 underwent surgery; the other five were managed conservatively [7]. Eighty-eight percent (45/51) of the patients were confirmed surgically and pathologically to have acute appendicitis. Abdominal pain, nausea, vomiting, leucocyte count, temperature and CRP had a low yield for acute appendicitis. Considerable pregnancy wastage occurred in those who underwent surgery in the first and second trimesters; the risk was higher among those who had perforated appendix. However, this study suffered from small sample size. Most evidence concerning presentation, management and outcome of appendicitis in pregnancy comes from case studies and retrospective series. Nevertheless, there is sufficient evidence for early intervention in suspected cases of acute appendicitis in pregnancy. In their small series of 10 patients, Mohan *et al*. reported significant differences in the rate of preterm labour (5.1% vs 1.3%) and foetal mortality (25% vs 1.7%) in patients with and without a perforated appendix [5].

The choice of interventions includes medical treatment with antibiotics and operative surgery, either laparotomy or laparoscopic appendicectomy [20]. Most surgeons opt for surgical intervention; the choice between laparotomy and laparoscopy is guided by resource availability and surgical skills. Delays in surgical intervention are associated with a higher rate of complications [2,7]. Masood *et al*. in a prospective cohort study of 118 patients who presented with appendicitis divided the cases into uncomplicated and complicated [10]. Statistically significant differences were obtained between the rates of operative time, postoperative fever, surgical site infections and poor obstetric outcomes in favour of uncomplicated appendicitis. Delays in diagnosis and intervention lead to complicated appendicitis, with resultant poorer surgical and pregnancy outcomes.

There are no robust data for success rates of HP after surgery; however, several case reports/series exist. Noor *et al*. reported a case of a ruptured HP who presented at 6 weeks of pregnancy and had an emergency laparotomy [14]. The patient went onto deliver a healthy live baby at 39 weeks. Jeon *et al*. reported a series of 48 cases from a single centre, who had HPs (1998-2012), following subfertility treatments [15]. Surgical treatment did not appear to affect rates of live births, with 80% of pregnancies progressing to live births. In most settings, data are absent or lacking because HPs are rare, necessitating long study periods. The outcome of our case is still to be determined, but pregnancy was continuing and viable at two-week review.

Clinical manifestations of HP include abdominal pain, vaginal bleeding and spotting. These symptoms are observed with intrauterine pregnancies as well. Serum β-hCG levels are not very helpful in the diagnosis of HP. Transvaginal ultrasound scan (TVS) is an important diagnostic modality. An IUP co-existing with an adnexal mass, gestational sac or ring sign can be visualised. However, TVS, may miss HP or may misdiagnose it as a corpus luteal cyst. In a report by Li *et al*. 58.93-73.75% of HPs were not confirmed before surgery [21]. Delayed diagnosis leads to an increased risk of intraperitoneal bleeding and hypovolaemic shock, with increased requirements for blood transfusion.

The surgical options for management of HP are laparotomy and laparoscopy. Medical management with methotrexate is not recommended due to well-documented teratogenicity. Eom *et al*. conducted a feasibility study of laparoscopic surgery for HP with obstetric outcomes as secondary outcomes [22]. Seventeen laparoscopic surgeries were performed, 14 for tubal and three for corneal HPs, 13 went on to deliver live babies, two had pregnancy wastage and outcomes for other two are not known. They demonstrated that laparoscopy is a feasible modality for HP, with no differences in outcomes. Li *et al*. analysed retrospectively the influence of different treatments on maternal and pregnancy outcomes [21]. The treatment modalities were expectant management, surgical management (laparotomy or laparoscopy) or transabdominal USS-guided TV aspiration of embryo (with or without selective fetocide). Sixty-four patients were followed between January 2003 and June 2014. Transabdominal USS-guided TV aspiration had the best maternal outcome and lowest abortion rate. Surgical management had the highest abortion rate whilst expectant management presented the worst maternal outcomes.

**Ethical considerations:** patient provided written informed consent for case write-up, use of images without identifying information and publication at 2-week review. The Joint Research Ethics Committee and the Medical Research Council of Zimbabwe do not require permission for publication of case reports.

## Conclusion

The simultaneous occurrence of a heterotopic pregnancy and acute appendicitis is a rare event that presents diagnostic and therapeutic challenges for clinicians and sonographers. Early surgical intervention by laparotomy or laparoscopy has the best maternal and pregnancy outcomes. This should be advocated for in suspected cases to reduce the morbidity, mortality and pregnancy wastage associated with delayed intervention.
